# A structural equation modeling approach to investigate HIV testing willingness for men who have sex with men in China

**DOI:** 10.1186/s12981-023-00561-9

**Published:** 2023-09-03

**Authors:** Han Jiang, Wei He, Haiying Pan, Xiaoni Zhong

**Affiliations:** School of Public Health, Chongqing Medical University, Chongqing, 400016 China

**Keywords:** MSM, HIV testing willingness, China

## Abstract

**Background:**

A substantial risk of contracting Human immunodeficiency virus (HIV) exists among men who have sex with men (MSM), and HIV infection rates have been rising. This study aimed to analyze the factors influencing the Chinese MSM population’s intention to test for HIV.

**Methods:**

Nonprobability sampling techniques were employed in June 2022 to recruit MSM in Chongqing and Sichuan, China. The data were analyzed using structural equation modeling (SEM), which is based on the knowledge-attitude-behavior (KAB) theory.

**Results:**

Among 1687 participants, 964 (57.1%) of the MSM were willing to have an HIV test. The results of the structural equation modeling (SEM) showed that knowledge, attitude, and behavior all influenced the testing intention, with attitude having the greatest impact (total effect of 0.22).

**Conclusion:**

HIV testing needs to be increased among MSM as they exhibit a moderate willingness to test. Improving education on HIV knowledge and risk behavior might enhance the willingness of MSM to test for HIV in China.

## Introduction

MSM communities are at high risk for HIV infection, with an infection risk that is 28 times greater than that of other adult men [1]. From 0.23 cases per 100,000 people in 2004 to 4.2 cases per 100,000 people in 2017, China has an upsurge in the prevalence of HIV [2]. For both public health detection and prevention, HIV testing is crucial. The effectiveness of routine HIV testing in lowering HIV infection rates in MSM populations has been demonstrated [3–6]. The main factors influencing the willingness to test for HIV in the MSM population are knowledge, sexual behavior, positive attitudes, and risk perception [7–10]. Additionally, HIV testing intention has a significant impact on the promotion of HIV testing among MSM, making this a worthwhile research issue [11]. Therefore, based on the KAB theory, we analyzed the influence of knowledge, attitude, and behavior on HIV testing willingness using SEM.

## Methods

### Research subjects

The non-probability sampling strategy was employed in this study to seek MSM volunteers in Chongqing and Sichuan through non-governmental organization (NGO) collaboration, peer referral and “snowballing” of core members, voluntary counseling and testing (VCT) clinics, and online channels like QQ and WeChat. After obtaining informed consent from the volunteers, a structured electronic questionnaire was distributed to the volunteers by the staff and then collected uniformly.

### Measures

Sociodemographic characteristics include age, household registration, ethnicity, educational level, employment status, marital status, and monthly disposable income. Based on previous literature, 13 observational variables were identified to measure knowledge of HIV [12]. Attitude was measured using four observed variables (i.e., “Getting tested for HIV helps people feel better”). Five variables were observed in behavior (i.e., drug usage).

### Statistical analysis

SAS version 9.4 was used for data collation and univariate analysis. Categorical data were described in frequencies and percentages, while continuous variables were expressed as means and standard deviations (SD). The chi-square test was used to compare differences between groups. The data were modeled and analyzed using MPLUS version 8.3 for structural equations. The variables with statistical significance (*p* < 0.05) were selected to construct the model.

## Results

1687 MSM in total met the study’s eligibility requirements. The participants’ average age was 29 (SD = 8.02) years old. A total of 964 (57.1%) MSM indicated a willingness to test in the next 6 months. Testing willingness varied by age, household registration, education level, employment status, and monthly disposable income (Table 1). The mean age of participants who were willing to test was 29.65(SD = 8.15), while those who did not intend to test for HIV in the future had a mean age of 27.85(SD = 7.71).

**Table 1:** Testing willingness of MSM with different demographic characteristics (n = 1687)

Variable	All	Willing to be vaccinated		*p*
		n	%	
N	1687	964	57.14	
Household registration				< 0.0001
Urban areas	1063	646	60.77	
Rural areas	624	318	50.96	
Ethnic groups				0.7836
Han nationality	1631	933	57.2	
Other ethnic minorities	56	31	55.36	
Highest education level				0.0089
Junior high and below	104	51	49.04	
High school/senior middle school/technical secondary school	349	180	51.58	
Junior college	511	292	57.14	
College and above	723	441	61	
Employment status				0.0004
Employed	1168	701	60.02	
Students	351	169	48.15	
Retired or unemployed	168	94	55.95	
Marital status				0.5383
Unmarried	947	536	56.6	
Married	664	380	57.23	
Divorced/widowed	76	48	63.16	
Monthly disposable income				0.0009
1000 RMB or less	125	57	45.6	
1001 ~ 3000 RMB	389	205	52.7	
3001 ~ 5000 RMB	547	311	56.86	
5001 ~ 10,000 RMB	515	316	61.36	
10,000 RMB or more	111	75	67.57	

The testing willingness of MSM with different knowledge, attitudes, behaviors is shown in Table 2. MSM who answered correctly were more likely to be tested for HIV than those who answered incorrectly. The vast majority of participants in our study had a positive view of HIV testing. For MSM, willingness to undergo HIV testing increases with the perceived benefits of HIV testing. Higher HIV testing intentions were reported by MSM with several sexual partners in the previous six months, no drug use, no commercial sex activity, and no STDs.

**Table 2:** Testing willingness of MSM with different knowledge, attitude, behavior. (n = 1687)

Variable	All	Willing to be vaccinated		*p*
		n	%	
**Knowledge**, n (%) answered correctly				
K1: If I eat with HIV-infected people, I will be infected with HIV.	1522	901	59.2	< 0.0001
K2: If I share needles with drug addicts, I will be infected with HIV.	1615	934	57.83	0.0067
K3: If I practice oral sex without condoms, I will be infected with HIV.	1224	698	57.03	0.8748
K4: Mosquito bites can cause HIV infection.	1263	776	61.44	< 0.0001
K5: If I use condoms correctly at each insertion, I can avoid HIV infection	1519	883	58.13	0.0137
K6: Removal of the penis from the vagina or anus before ejaculation can prevent HIV infection.	1304	770	59.05	0.0035
K7: Being with only one uninfected loyal partner can prevent HIV infection.	1196	705	58.95	
K8: All HIV-infected pregnant women give birth to HIV-infected children.	968	601	62.09	< 0.0001
K9: People who use antibiotics are not infected.	1430	847	59.23	< 0.0001
K10: Examination results at 1 week after sexual intercourse can determine whether the person is infected with HIV.	656	451	68.75	< 0.0001
K11: AIDS can not be cured.	1424	841	59.06	0.0002
K12: Oral sex is much less likely to transmit HIV than anal intercourse.	736	414	56.25	0.5145
K13: The risk of HIV infection can be reduced by the treatment of sexually transmitted diseases.	1284	735	57.24	0.8821
**Attitude**, n (%) answered agree				
A1: Getting tested for HIV helps people feel better	1460	896	61.37	< 0.0001
A2: Getting tested for HIV helps people avoid HIV infection	1438	894	62.17	< 0.0001
A3: Regular HIV antibody testing is necessary	1417	908	64.08	< 0.0001
A4: The institutions nearby offer reliable HIV antibody testing services **Behavior (last 6 months)**	1329	834	62.75	< 0.0001
B1: Number of male sexual partners				< 0.0001
None	527	226	42.88	
1	868	547	63.02	
2 or more	292	191	65.41	
B2: Commercial sex activity				< 0.0001
Never	1466	873	59.55	
Done	221	91	41.18	
B3: Frequency of searching for sexual partners through the internet				0.0002
Never	1066	604	59.66	
Sometimes or occasionally	481	300	62.37	
Frequently	140	60	42.86	
B4: Drug use				< 0.0001
Never	1550	920	59.35	
Done	137	44	32.12	
B5: History of STD				< 0.0001
No	1404	843	60.04	
Yes	283	121	42.76	

### Structural equation model

**Fig. 1:**
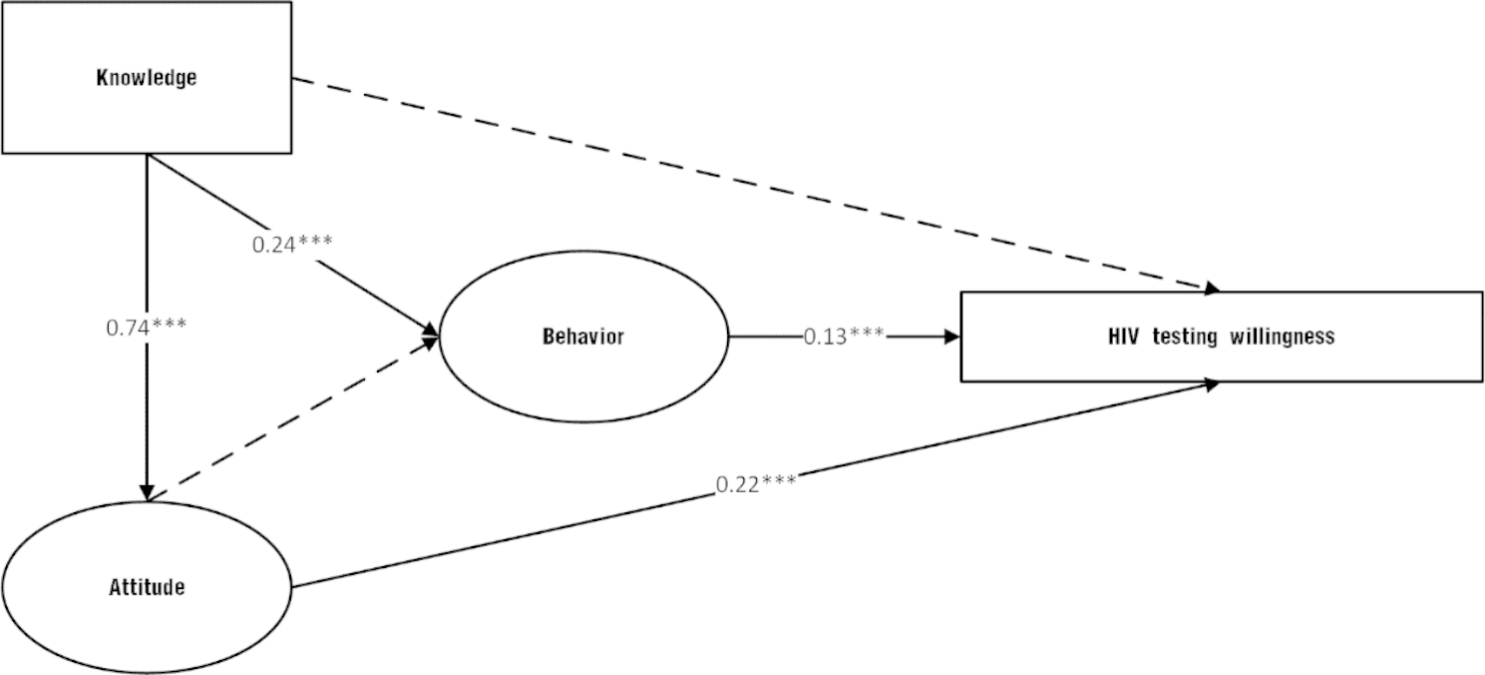
Modified structural equation model of HIV testing willingness. Note: A solid line indicates that the relationship is statistically significant (*p* < 0.05), and a dashed line indicates that the relationship had no statistical significance (*p* > 0.05). *** indicated statistical significance with *p* < 0.001

Figure 1 presents the results from the modified structural equation modeling. The fit indices show that the hypothesized model fit the data acceptably well (CFI = 0.99, TLI = 0.99 RMSEA = 0.04, SRMR = 0.02, and Chi-square/df = 4.2). Knowledge affected HIV testing willingness mainly by changing attitudes, and the standardized indirect effect was 0.19. The standardized direct effect of attitude on willingness to test for HIV was 0.22. The direct effect of behavior on HIV testing willingness was 0.13.

## Discussion

Our study found that MSM had a moderate level of willingness to participate in HIV testing. 57.1% of MSM expressed willingness to be tested for HIV in the next 6 months. A high level of HIV-related knowledge could help MSM establish a correct HIV risk perception and make them aware of the benefits of HIV testing, thus increasing their willingness to test for HIV, according to structural equation modeling. The positive effect of MSM’s HIV knowledge on willingness to test was primarily indirect through its effect on attitude, the study found. This was in line with earlier research, which found that higher levels of knowledge were strongly linked to more optimistic attitudes and that MSM who were more aware of HIV/AIDS were more likely to be open to getting tested for the virus [13, 14]. It is recommended that the government actively take effective measures to strengthen HIV knowledge and enrich the content of HIV knowledge so that high-risk groups with relatively low knowledge can understand the necessity and importance of HIV testing.

Attitude had the greatest impact on willingness to test for HIV. Our findings showed that MSM with a greater perceived benefit of HIV testing had a higher willingness to test. Positive, accurate HIV testing beliefs can, to some extent, influence MSM’s HIV testing habits. But the development of beliefs must take place over time. In order for high-risk people to acquire positive and accurate concepts to raise testing rates, national authorities should continue to push knowledge about the necessity, importance, and benefits of HIV testing.

Consistent with previous findings, our study showed that MSM with high-risk sexual behaviors have a lower willingness to test for HIV [15–17]. As a result, MSM who engage in hazardous sexual activity and are sexually active should be the focus of HIV prevention education, which is essential for lowering HIV incidence. There were limitations to this study. Firstly, this study asked about sensitive topics and behaviors in the past 6 months, which may have been affected by reporting bias and recall bias. Secondly, because participants were recruited in Chongqing, Sichuan, the obtained results may not be generalizable to all MSM with HIV.

## Conclusion

In our study, more than half of the MSM agreed to have an HIV test. We suggested that a series of measures targeting HIV awareness, high-risk sexual behavior, and attitude development should be taken to increase the willingness of the MSM population to test for HIV.

## Data Availability

The datasets involved in the current study are not publicly available due to privacy but are available from the corresponding authors.

## Abbreviations

HIV: human immunodeficiency virus
MSM: men who have sex with men
WHO: world health organization
SEM: structural equation modeling
KAB: knowledge-attitude-behavior theory
NGO: non-governmental organization
AIDS: acquired immunodeficiency syndrome
VCT: voluntary counseling and testing
STD: sexually transmitted diseases
ML: maximum Likelihood
SD: standard deviation
RMSEA: root mean square error of approximation
TLI: tucker-lewis index
CFI: Comparative Fit index
SRMR: Standardized Root Mean Square Residual
